# Diammonium Hydrogen Citrate-Assisted Spray Pyrolysis Synthesis of Nanostructured LiCoPO_4_ Microspheres as High-Voltage Cathode Material for Lithium-Ion Batteries

**DOI:** 10.1021/acsomega.4c03752

**Published:** 2024-09-13

**Authors:** Ayaulym Belgibayeva, Takeru Nagashima, Wenyu Cui, Daiki Sueyoshi, Izumi Taniguchi

**Affiliations:** † Department of Chemical Science and Engineering, Tokyo Institute of Technology, Tokyo 152-8552, Japan; ‡ National Laboratory Astana, Nazarbayev University, Kabanbay Batyr Ave. 53, Astana 010000, Kazakhstan

## Abstract

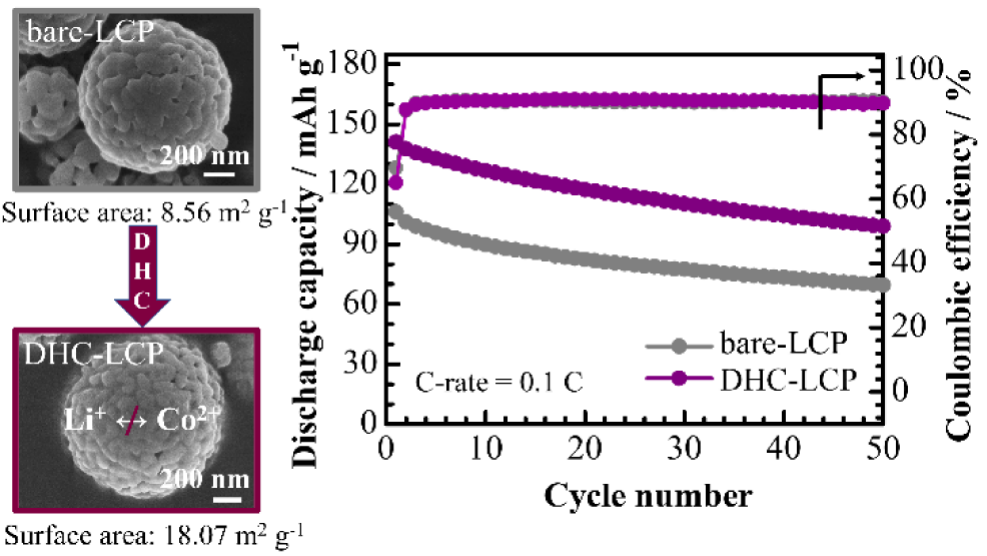

Nanostructured LiCoPO_4_ (LCP) microspheres were successfully synthesized by one-step spray pyrolysis, adding an appropriate amount of diammonium hydrogen citrate (DHC) additive to the precursor solution. Comprehensive physical characterization confirmed that the obtained LCPs exhibited a desirable orthorhombic olivine structure with nanostructured morphology and a significant increase in specific surface area. This enhancement was attributed to the dispersion effect due to the carboxyl group and the evolution of the ammonium group of DHC during the pyrolysis process. The resultant LCP delivered a high initial discharge capacity of 132 mA h g^–1^ with 63.3% capacity retention (vs 103 mA h g^–1^ and 37.1% of bare-LCP) after 50 cycles at 0.1 C using the conventional electrolyte. Moreover, the electrochemical performance showed additional enhancement when a fluorinated electrolyte was introduced, resulting in initial and 50th discharge capacities of 141 and about 100 mA h g^–1^, respectively, at 0.1 C.

## 1 Introduction

Lithium-ion batteries (LIBs) have revolutionized portable electronics, electric vehicles, and grid energy storage due to their high energy density, long cycle life, and relatively low self-discharge rate.^1,2^ The development of high-performance cathode materials is crucial for further enhancing the performance of LIBs, especially in terms of energy density, safety, and environmental sustainability.^3,4^ The energy density of a battery can be enhanced through two primary methods: by augmenting the practical capacity or by elevating the operating potential of the cathode materials while maintaining consistent masses. Olivine-type LiCoPO_4_ (LCP) emerges as a promising candidate for high-voltage cathode materials in the next-generation LIBs, exhibiting remarkable electrochemical properties such as a high operating potential of ∼4.8 V vs Li/Li^+^ and a moderate theoretical capacity of ∼167 mA h g^–1^.^5,6^ However, the translation of these favorable characteristics into practical applications is hindered by various challenges, including limitations in achieving high Coulombic efficiency, robust rate capability, and extended cycle stability.^7,8^ These obstacles are largely attributed to factors such as low electronic and ionic conductivities, the formation of antisite defects or Li–Co cation mixing within the crystal structure of LCP during the synthesis process, as well as decomposition of the conventional electrolytes at high operating voltages.^9,10^

While the issue of electrolyte compatibility can be effectively addressed by incorporating oxidation-resistant fluorinated cosolvents like fluoroethylene carbonate (FEC), there are relatively few studies examining the electrochemical performance of LCP with such electrolytes.^11,12^ Conversely, considerable efforts have focused on overcoming other structural challenges of LCP, particularly concerning morphology and aiming to reduce lithium-ion diffusion pathways, enhance electrode–electrolyte contact, and improve structural stability during cycling.^13−18^ Various strategies have been explored, including incorporating chelating/dispersing organic additives and surfactants, as well as controlling precursor compositions and employing diverse synthesis methods.^19−21^ Among these strategies, spray pyrolysis stands out as a versatile technique for the scalable production of functional materials, allowing precise control over composition, morphology, and structure.^17,18,22−24^

Previous studies have demonstrated that despite numerous attempts to control the morphology, achieving high performance in LCP electrodes often relies on additional carbon coating.^18,19,25,26^ However, this process introduces electrochemically inactive weight to the electrode material, leading to a reduction in the overall energy density. Additionally, it often necessitates additional synthesis steps such as ball-milling and further sintering to maintain both the nanostructure and high crystallinity of LCP while ensuring uniform carbon coating.^16,17^ Unfortunately, these additional steps contribute to the decreased energy efficiency in the overall synthesis process. Hence, from an engineering perspective, a one-step synthesis process capable of producing high-performance LCP without the need for an additional carbon coating presents significant advantages. Recently, Li and Taniguchi introduced a one-step synthesis method for producing high-performance spherical nanostructured LCP particles using citric acid (CA)-assisted spray pyrolysis.^24^

Another approach for controlling the nanostructure of cathode materials involves introducing N-containing compounds that evolve during the pyrolysis process.^27^ However, diammonium hydrogen citrate (DHC) could potentially serve as an additive that combines both features (dispersion effect and ammonium evolution), and to the best of our knowledge, its effect on the physical and electrochemical properties of LCP has not been reported yet.

In this work, nanostructured LCP microspheres were synthesized by one-step ultrasonic spray pyrolysis using a DHC additive (denoted as DHC-LCP) and applied as cathode material for LiBs. Physical and electrochemical properties of the prepared material were studied in comparison with those synthesized using a CA additive (CA-LCP) and without any additives (bare-LCP) to elucidate the effect of ammonium and carboxyl groups on the morphology of LCP. Additionally, a fluorinated electrolyte was utilized to suppress the electrolyte-related cell degradation and further highlight the effect of the DHC additive on the electrochemical performance of the developed LCP materials.

## 2 Experimental Section

### 2.1 Synthesis of Materials

Nanostructured LCP microspheres were prepared using one-pot ultrasonic spray pyrolysis^28^ at 600 °C and an air flow rate of 2 L min^–1^, as reported elsewhere^24^ by changing Li and P sources. The precursor solutions contained stoichiometric amounts of LiNO_3_, Co(NO_3_)_2_·6H_2_O, and H_3_PO_4_ dissolved in distilled water with a total concentration of 0.12 M (denoted as bare-LCP) and DHC or CA additives at 0.008 M (denoted as DHC-LCP or CA-LCP, respectively). All chemicals mentioned above were purchased from Wako Pure Chemical Industries Ltd., Japan.

After spray pyrolysis, the as-prepared powders were dried at 110 °C for 6 h in a vacuum oven to remove the moisture.

### 2.2 Physical Characterizations

The crystalline phase of the samples was identified by X-ray powder diffraction (XRD, Rigaku, Ultima IV with D/teX Ultra) analysis with Cu Kα radiation (λ = 1.5406 Å) at 40 kV, 40 mA, and a scan rate of 5° min^–1^ ranging from 10° to 70°. The lattice parameters of the materials and antisite defect concentrations were refined by Rietveld analysis with the integrated XRD software package PDXL2 (Rigaku) and FullProf Suite, respectively. The crystallinity of the samples was estimated by the full width at half maximum (fwhm) value of the (311) plane peak of the XRD patterns, which were calculated by the same software using Scherrer’s formula. Fourier-transform infrared (FT-IR) spectroscopy (Shimadzu IRAffinity-1 Miracle-10 FT-IR spectrometer) was used to identify the chemical bonds in the prepared samples. X-ray photoelectron spectroscopy (XPS) was used to clarify the chemical bonds and valence states of elements within the prepared samples (KRATOS ULTRA2, Shimadzu). The spectrum analysis conditions were as follows: X-ray: 300 W (monochromatic AlKα); pass energy: wide 160 eV, narrow 20 eV; analysis diameter: 700 × 300 μm; charge neutralizer: on.

The particle morphology of the samples was examined by scanning electron microscopy (SEM; KEYENCE, VE-9800) at 20 kV. Because of the low electroconductivity of samples, the platinum coating was conducted in advance using a sputtering facility (ELIONIX, ESC-101). Field emission scanning electron microscopy (FE-SEM; HITACHI, SU9000) was applied for high-resolution observation at 8 kV. The theoretical 2.5 mm osmium coating was achieved by an osmium plasma coater (Meiwafosis, Neoc). For internal structure observation, a cross-section polisher (CP; JEOL, SM-09020CP) was used for the sample preparation. Thermal decomposition behavior of DHC was evaluated using thermogravimetric analysis coupled with differential thermal analysis (TG-DTA, Rigaku, TG-8120) in air with a heating rate of 10° min^–1^.

The specific surface area of the samples was determined by the Brunauer–Emmett–Teller (BET) method from N_2_ absorption–desorption isotherms. The nitrogen adsorption–desorption measurements were performed at a liquid N_2_ temperature of 77 K by using a Micromeritics TriStar II 3020 system.

### 2.3 Electrochemical Characterizations

Electrochemical characterization was performed by assembling a CR2032 coin-type cell for galvanostatic charge–discharge testing. The cell comprised a lithium metal negative electrode and LCP sample positive electrode that were separated by a microporous polypropylene film. A 1 M solution of LiPF_6_ in a mixed solvent of ethylene carbonate (EC) and dimethyl carbonate (DMC) with a 1:1 volume ratio (Tomiyama Pure Chemical Co., Ltd.) was used as the conventional electrolyte and 1 M LiPF_6_ in FEC:DMC = 1:4 (vol) was used as the fluorinated electrolyte. To ensure proper wettability, the separators were soaked in the electrolytes for 1–2 weeks, and an excess amount of electrolyte (about 100 μL) was added during the cell assembling. The cathode consisted of 70 wt % LCP, 10 wt % poly(vinylidene fluoride) (PVdF) as a binder, and 20 wt % acetylene black (AB). These materials were dispersed in 1-methyl-2-pyrrolidinone (NMP), spread uniformly onto an aluminum foil using the doctor blade technique, and then dried in a vacuum oven for 4 h at 110 °C. The cathode was punched into circular discs and then scraped in order to standardize the area of the cathode (1 cm^2^). The cell was assembled inside a glovebox filled with high-purity argon gas (99.9995% purity).

The cells were tested galvanostatically in the potential range of 3.5–5.3 V vs Li/Li^+^ on multichannel battery testers (Hokuto Denko, HJ1010mSM8A) at different charge–discharge rates from 0.1 to 1 C (1 C = 167 mA g^–1^). Current densities and specific capacities were calculated on the basis of the mass of LCP in the cathode. The mass loading of the active materials was 1.0 ± 0.1 mg cm^–2^.

Cyclic voltammetry (CV) measurements were performed in the potential range of 3.5–5.3 V vs Li/Li^+^ at a scanning rate of 0.1 mV s^–1^, and electrochemical impedance spectroscopy (EIS) measurements were carried out at the amplitude of the AC signal of 10 mV in the frequency range from 100 kHz to 0.01 Hz, using a VMP3B-5 potentiostat/galvanostat (Bio-Logic SAS, SP-150). All of the electrochemical measurements were performed at room temperature.

## 3 Results and Discussion

### 3.1 Effect of Additives on the Formation of Nanostructured LCP Microspheres

The structural and morphological characteristics of the as-prepared LCP samples were analyzed to elucidate the impact of additives and their relationship to electrochemical properties as cathode materials for LIBs.

**Figure 1:**
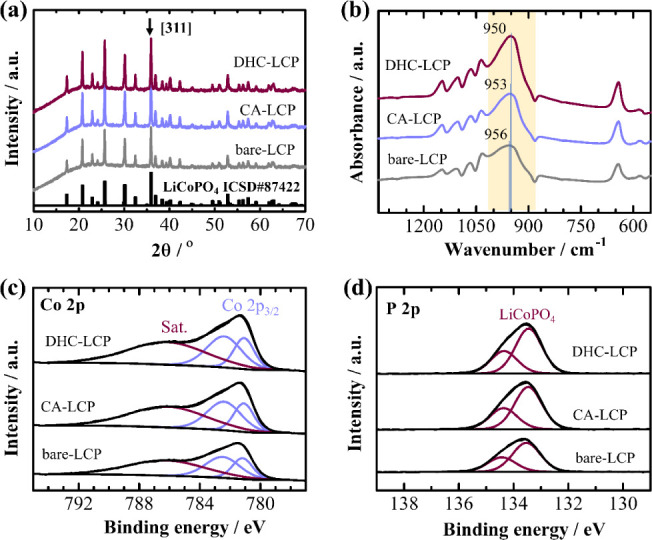
XRD patterns (a), FT-IR spectra (b), Co 2p (c), and P 2p (d) XPS spectra of LCP samples.

Figure 1a shows the XRD patterns of the LCP samples prepared with and without different additives. All peaks observed in the XRD patterns of the three samples are attributed to the orthorhombic olivine structure and exhibit the *Pnma* space group. However, very weak Li_3_PO_4_ peaks were observed around 20° in the detailed XRD analysis (Figure S1). After the introduction of additives, only minor changes in the lattice parameters were observed, indicated by a small decrement, as summarized in Table 1. Conversely, an enhancement in crystallinity was noted with the inclusion of additives, demonstrated by the fwhm value of the (311) reflection, as detailed in Table 2. The increase in crystallinity indicates improved ordering and alignment of crystal planes within the LCP structure. Notably, the DHC-LCP sample demonstrated the highest level of crystallinity among the tested samples. This enhancement may lead to enhanced electrochemical properties, such as improved charge–discharge kinetics and cycling stability, owing to the more ordered and well-defined crystal structure.^9^

**Table 1:** Lattice Parameters of LCP Prepared without and with Different Additives

Name	*a*/Å	*b*/Å	*c*/Å	*V*/Å^3^
bare-LCP	10.2070	5.9260	4.7047	284.5740
CA-LCP	10.2066	5.9231	4.7037	284.3618
DHC-LCP	10.2052	5.9236	4.7039	284.3557
LCP (ref ICSD#84722)	10.2020	5.9227	4.7004	284.0141

**Table 2:** Crystallinity Information (fwhm) and Specific Surface Area of LCP Prepared without and with Different Additives

Name	fwhm [311]	BET specific surface area, m^2^ g^–1^
bare-LCP	0.154	8.56
CA-LCP	0.185	14.17
DHC-LCP	0.190	18.07

FTIR analysis was conducted to characterize antisite defects in the LCP samples and is depicted in Figure 1b. Upon the addition of additives, a notable red shift was observed in the PO_4_ band, which typically manifests between 900 and 1000 cm^–1^ in the FTIR spectra. This specific band is particularly sensitive to the exchange of lithium and cobalt ions within adjacent LiO_6_ and CoO_6_ octahedra. The observed red shift indicates a decrease in the concentration of antisite defects within the crystal lattice of the LCP samples.^9^ Interestingly, the red shift was most prominent in the DHC-LCP sample, suggesting a significant reduction in antisite defect concentration compared with other samples. This observation highlights the effectiveness of DHC as an additive in minimizing structural defects in the LCP material. As confirmed from Rietveld refinement of XRD patterns (Figure S2), the concentration of antisite defects was lowered almost twice in DHC-LCP compared to bare-LCP, indicating a more ordered and stable crystal structure, which can positively impact the electrochemical performance of the material in LIBs.

Given the relatively high temperature (600 °C) of spray pyrolysis in air, it is crucial to ensure that LCP is not oxidized. The chemical bonds and valence states of elements within the LCP samples were further investigated through XPS, as illustrated in Figure 1c,d. In the Co 2p XPS spectra of all three samples, the signals split into 2p_3/2_ and 2p_1/2_ multiplets. Specifically, the 2p_3/2_ peak exhibited a triple structure with peaks located at 781.18, 782.49, and 786.22 eV, which is consistent with previous observations for similar LCP materials.^13^ The primary peak binding energy aligns closely with that of CoF_2_ (783.0 eV), confirming an oxidation state of +2 for the surface Co atoms. The depletion of the Co^2+^ peak could be attributed to the possible differences in the bonding. Despite minor variations in peak positions, the ratio of these peaks remains consistent across all three samples (Table S1). It is worth mentioning that no peaks of Co^3+^, which are typically found at around 780 eV, were observed.^29^ Analysis of the P 2p spectra across all three samples, which includes the 2p_3/2_ and 2p_1/2_ doublet, revealed a dominant component appearing within the range of 133.5–134.4 eV. This observation is in line with the phosphate bond present in LCP, indicating an oxidation state of +5 for phosphorus.^14^

**Figure 2:**
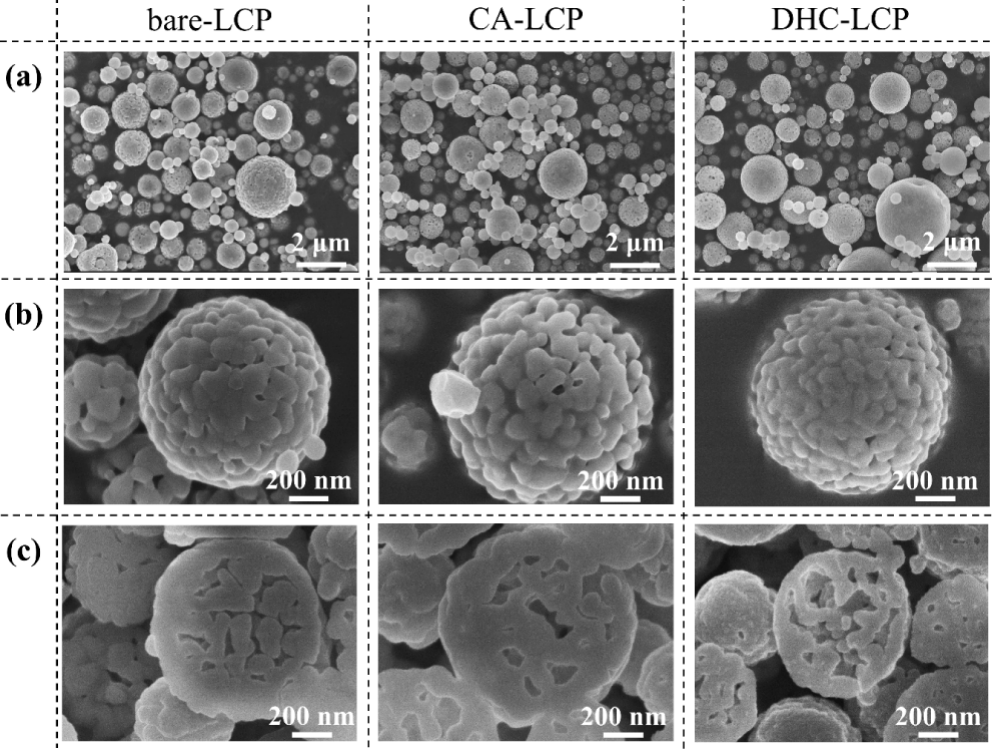
Surface SEM images at different magnifications (a and b) and cross-sectional SEM images (c) of LCP samples.

The morphology of samples was characterized by SEM and is shown in Figure 2. Notably, all three samples exhibited similar shapes and secondary particle sizes, as evidenced by Figure 2a. However, upon the introduction of additives, a noticeable reduction in primary particle size was observed, with the DHC-LCP sample showcasing the finest nanostructure. This was further characterized by a rough surface texture with small grooved patterns indicative of higher porosity, as illustrated in Figure 2b. This enhanced porosity contributed to the high BET specific surface area of DHC-LCP, which was found to be more than twice that of bare-LCP (Table 2), suggesting an increased accessibility of active sites for electrochemical reactions. The observed reduction in primary particle size and increased porosity in DHC-LCP can be attributed to several factors. First, the dispersion effect of additives may have restricted the agglomeration of primary particles during synthesis. Additionally, the evolution of ammonium from DHC during pyrolysis could have influenced the nanostructure formation process, confirming the advantage of DHC over CA as nanostructure-controlling additive.

Furthermore, the cross-sectional images depicted in Figure 2c revealed a similar patterned and rough inner wall structure in all three samples. This observation suggests a consistent inner morphology across the samples and highlights the potential of this method for convenient morphological modulation of micro- and nanospheres.

**Figure 3:**
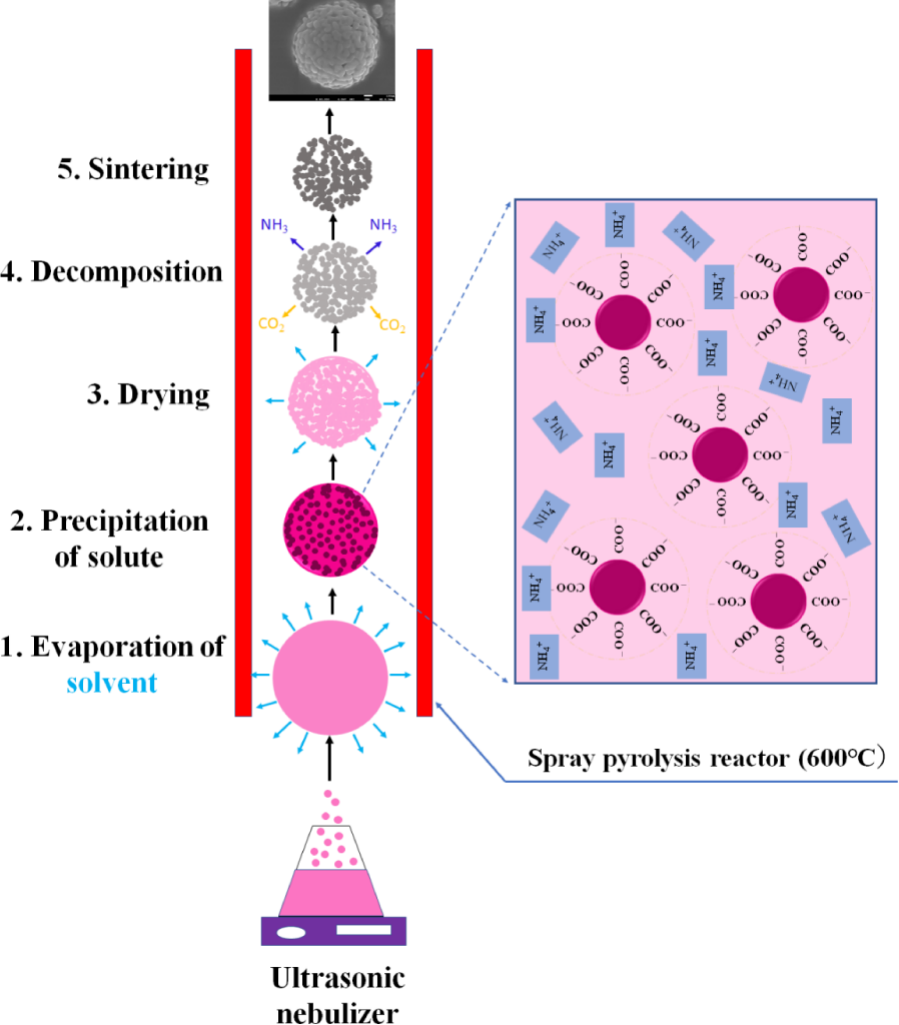
Schematic illustration of the formation of nanostructured LCP microspheres during the spray pyrolysis process.

Figure 3 illustrates the possible mechanism behind the formation of nanostructured LCP microspheres. Droplets generated by an ultrasonic nebulizer are carried into the quartz reactor tube heated at 600 °C by air. The droplets are heated, and then, the evaporation of solvent (1) and precipitation of solute (metal phosphates) (2) subsequently proceed. During the precipitation process, carboxyl groups of DHC are adsorbed on the surface of precipitated particles, and they are negatively charged, confining their agglomeration. As drying (3) and decomposition (4) progresses, DHC, as a source of ammonium and carboxyl groups, undergoes a multistep decomposition process in air (Figure S3) with two main mass losses centered around 200 and 550 °C, decomposing with generation of NH_3_ and CO_2_ gases almost completely by 600 °C. As a result, further agglomeration of the precipitated particles is suppressed during the sintering process (5). These processes create channels within the secondary particles, forming the internal structure of the patterned design in the DHC-LCP samples.

### 3.2 Effect of Additives on the Electrochemical Properties of Nanostructured LCP Microspheres

**Figure 4:**
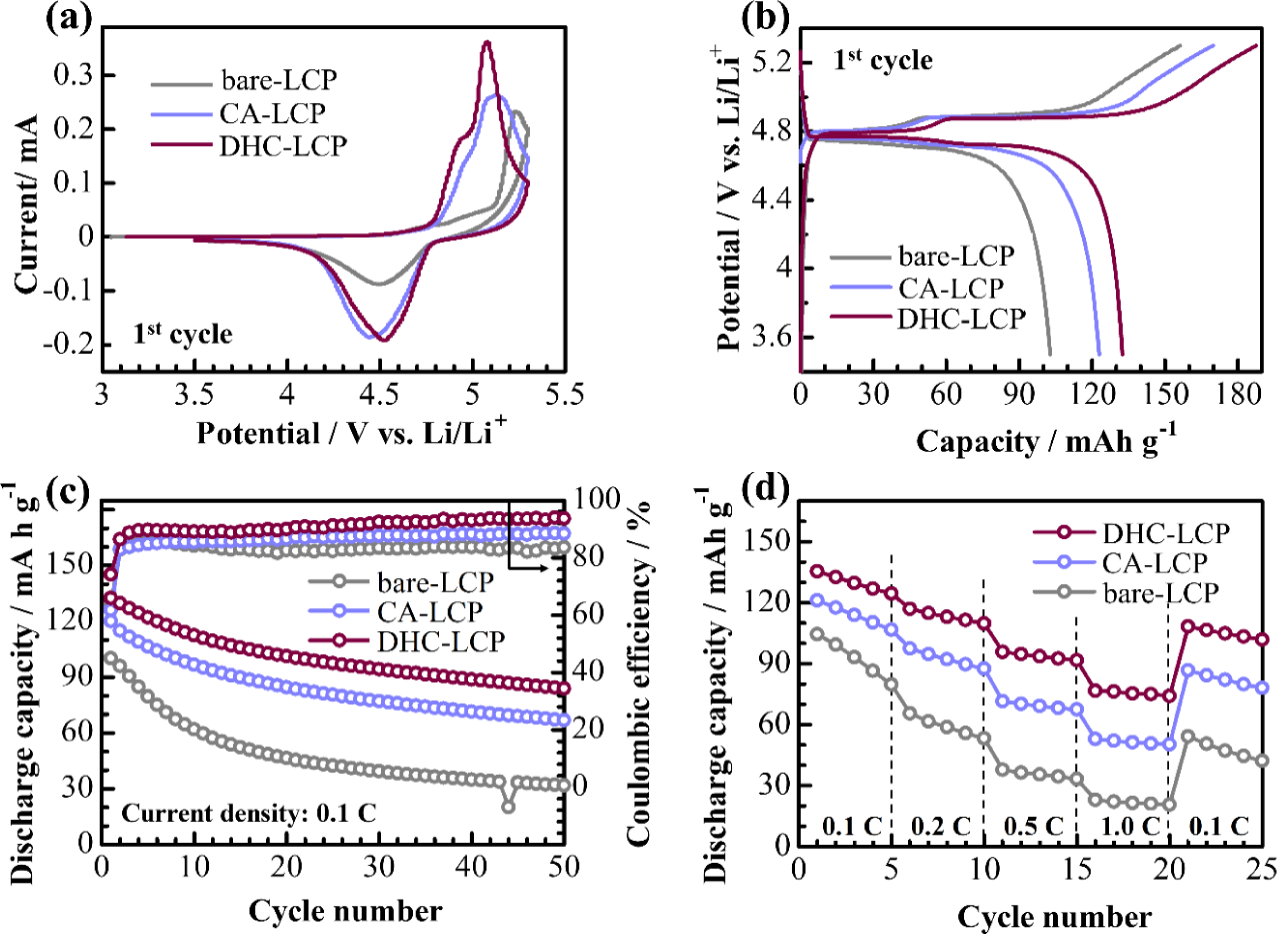
Initial CV curves (a), initial potential profiles (b), cycle performance at 0.1 C (c), and rate-capability (d) of the LCP samples with the conventional electrolyte.

Figure 4a presents the CV curves of the LCP samples, offering comprehensive insight into the effect of additives on the electrochemical behavior. Notably, DHC-LCP stands out with its prominently intensified oxidation–reduction peaks, indicative of enhanced kinetics during the delithiation–lithiation processes. This heightened intensity suggests that DHC-LCP possesses superior charge transfer properties and facilitates more efficient ion insertion–extraction compared to other samples. Furthermore, the minimal difference observed in the positions of the oxidation–reduction peaks implies a reduced overpotential in DHC-LCP, which is crucial for minimizing energy losses during charging–discharging cycles and improving overall battery efficiency. This reduced overpotential signifies that DHC-LCP can achieve higher energy conversion efficiency, contributing to its superior electrochemical performance.

The initial charge–discharge curves in Figure 4b further underscore the exceptional performance of DHC-LCP, revealing its highest first discharge capacity of 132 mA h g^–1^ at 0.1 C, which surpasses that of CA-LCP (123 mA h g^–1^) and bare-LCP (103 mA h g^–1^). This substantial enhancement in discharge capacity signifies the superior lithium storage capability of DHC-LCP, which can accommodate more lithium ions during the discharge process, leading to higher energy density and improved battery performance.

The cyclability data in Figure 4c illustrate the long-term stability of DHC-LCP, with the highest capacity retention rate of 63.3% after 50 cycles at 0.1 C, compared to 55.6% for CA-LCP and 31.7% for bare-LCP. This remarkable capacity retention indicates the robustness and durability of DHC-LCP over repeated charge–discharge cycles, highlighting its potential for long-term use in practical battery applications. The Coulombic efficiencies of the samples after 10 cycles follow the same trend as the observed specific discharge capacities, with DHC-LCP achieving the highest Coulombic efficiency of 94% after 50 cycles. This could be attributed to the decreased overpotential in cells with DHC-LCP, which allows high-voltage delithiations of LCP with minimal impact on electrolyte decomposition. Moreover, Figure 4d shows the outstanding rate-capability of DHC-LCP, as it maintains the highest discharge capacity of 75 mA h g^–1^ even at a high current density of 1 C. This superior rate capability is crucial for applications requiring rapid charging–discharging, such as electric vehicles and portable electronics, where maintaining a high energy output at high currents is essential.

**Figure 5:**
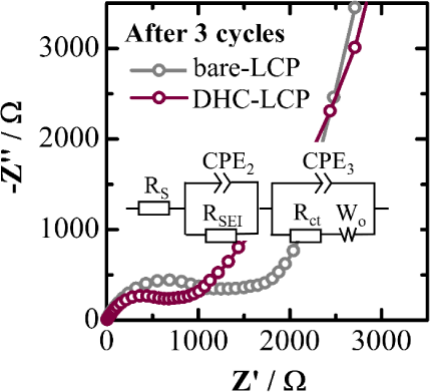
Nyquist plots of the LCP samples with conventional electrolyte after 3 cycles. Inset: equivalent circuit model.

Figure 5 compares the Nyquist plots of the LCP samples prepared with and without the DHC additive after three cycles. Both spectra exhibit a prolonged semicircle, comprised of two depressed semicircles in the high- to medium-frequency regions, as well as a straight line in the low-frequency region. These features correspond to the impedance components of solid electrolyte interphase (*R*_SEI_), charge transfer (*R*_CT_), and the diffusion of lithium ions into the electrode (or Warburg impedance), as indicated in the equivalent circuit model in the inset of Figure 5.^24^ The incorporation of DHC into the high-voltage LCP cathode results in a nearly 2-fold decrease in both the *R*_SEI_ and *R*_CT_, as summarized in Table 3. These impedance reductions play a pivotal role in enhancing cyclability and rate-capability. These results highlight the remarkable performance of DHC-LCP as a cathode material for LIBs. Its enhanced kinetics, reduced overpotential, high discharge capacity, excellent cyclability, and superior rate capability collectively demonstrate the significant advantages conferred by the incorporation of DHC as an additive, positioning DHC-LCP as a promising candidate for high-performance energy storage devices.

**Table 3:** Fitting Results of the Nyquist Plots of LCP Samples with a Conventional Electrolyte

Sample	*R*_s_/Ω	*R*_SEI_/Ω	*R*_CT_/Ω
bare-LCP	6.0	442.6	735.1
DHC-LCP	8.6	247.6	401.9

### 3.3 Electrochemical Properties of Nanostructured LCP Microspheres with Fluorinated Electrolyte

**Figure 6:**
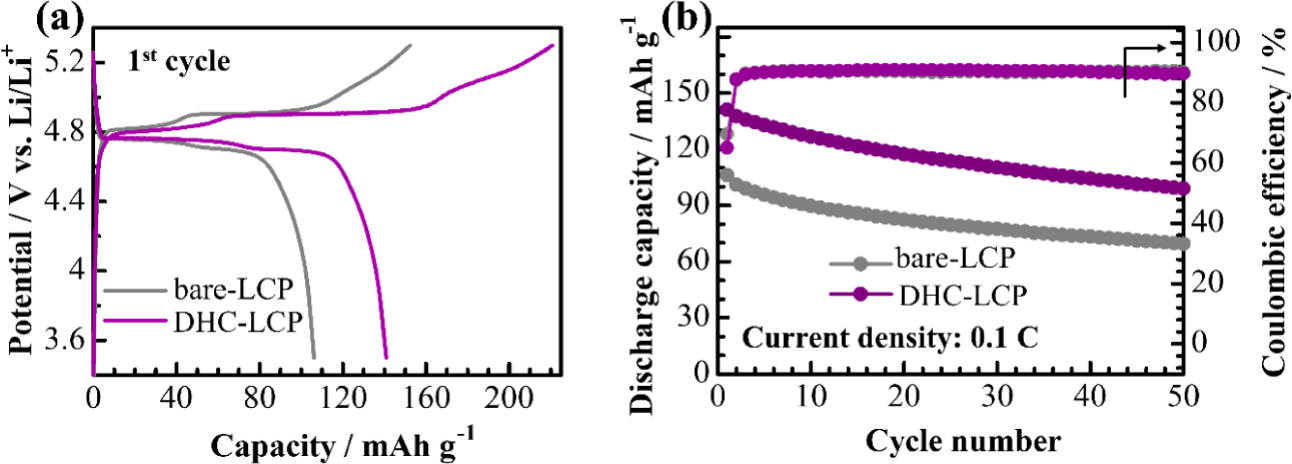
Initial potential profiles (a) and cycle performance at 0.1 C (b) of the LCP samples with a fluorinated electrolyte.

A fluorinated electrolyte (1 M LiPF_6_ in FEC:DMC = 1:4 (vol)) was further employed to mitigate cell degradation caused by electrolyte-related issues, allowing for a clearer assessment of the impact of the DHC additive on the electrochemical performance of the developed LCP material. According to the results presented in Figure 6a, upon the introduction of the fluorinated electrolyte, the DHC-LCP sample demonstrated a substantial increase in initial discharge capacity, reaching 141 mA h g^–1^ at 0.1 C, while the capacity of the bare-LCP remained relatively unchanged. Additionally, both samples exhibited more distinct charge–discharge plateaus compared to those observed with conventional electrolytes in Figure 4b, indicating improved electrochemical kinetics and enhanced charge transfer within the electrodes. This improvement translated into enhanced cycling stability for both DHC-LCP and bare-LCP samples, maintaining discharge capacities of about 100 and 70 mA h g^–1^, respectively, after 50 cycles at 0.1 C, as illustrated in Figure 6b. Remarkably, both samples demonstrated more stable Coulombic efficiency with the fluorinated electrolyte compared with the conventional electrolyte (Figure 4c), retaining 90% of their charge–discharge efficiency throughout the cycling process. These results underscore the significant potential of DHC additive-assisted synthesis in enhancing the electrochemical performance and stability of LCP-based materials, particularly when combined with fluorinated electrolytes.

The improved electrochemical characteristics of DHC-LCP can be attributed to several key factors: increased crystallinity, a reduced concentration of antisite defects, a nanostructured morphology characterized by a smaller primary particle size, and a higher specific surface area. These properties collectively facilitate improved electrolyte penetration, increased contact area, greater availability of electrochemically active sites, enhanced structural stability, and shortened diffusion paths for lithium ions within the cathode material. Overall, the introduction of DHC represents a promising strategy for enhancing the performance of high-voltage LCP cathodes in LIB applications.

**Figure 7:**
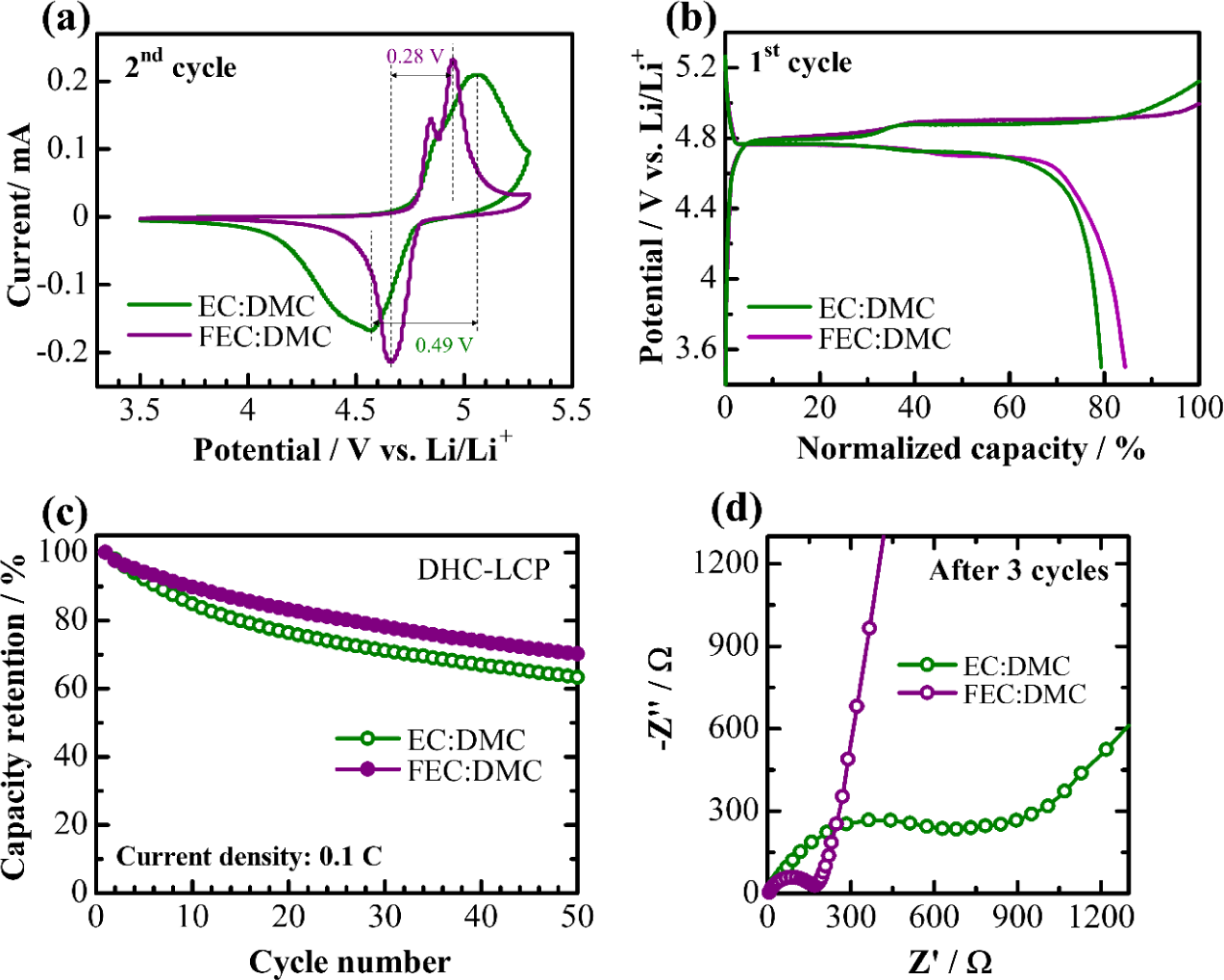
CV curves for the second cycle (a), normalized initial galvanostatic charge–discharge profiles (b), capacity retention over 50 cycles at 0.1 C (c), and Nyquist plots of DHC-LCP cells with conventional and fluorinated electrolytes after three cycles (d).

The effect of the fluorinated electrolyte on the electrochemical performance of the DHC-LCP was further studied in comparison to conventional electrolyte and is presented in Figure 7. CV curves for the second cycle of cells containing DHC-LCP with both conventional and fluorinated electrolytes in Figure 7a exhibit notable differences, with the fluorinated electrolyte demonstrating more distinct oxidation–reduction peaks compared to those of the conventional electrolyte, indicating a more efficient and reversible redox process. Additionally, the fluorinated electrolyte shows reduced peak splitting and lower overpotential (0.28 V vs 0.49 V), suggesting improved kinetics and reduced energy barriers for the electrochemical reactions.

In the normalized galvanostatic charge–discharge profiles (100% corresponds to the theoretical capacity of 167 mA h g^–1^), the potential plateaus with the fluorinated electrolyte are more distinct and extended, with a length increase of approximately 5%, as depicted in Figure 7b. This elongation translates to a higher utilization of the active material, achieving 85% of the theoretical capacity during the initial cycle. This improvement suggests that the fluorinated electrolyte enhances the electrochemical environment, allowing for more complete lithiation and delithiation processes. Moreover, Figure 7c shows that cells with the fluorinated electrolyte retain about 10% more capacity compared to those with the conventional electrolyte after 50 cycles at 0.1 C. These improvements can be primarily attributed to the formation of a stable and conductive LiF-rich SEI layer, which facilitates efficient lithium-ion transport and mitigates degradation processes.^11,12^ Moreover, as confirmed from the digital images of the surface of Li metal after 10 cycles at 0.1 C with different electrolytes (Figure S4), the fluorinated electrolyte prevents the dissolution and migration of reaction products toward the negative electrode during cycling.

According to Nyquist plots in Figure 7d, the overall resistance in cells with the fluorinated electrolyte is four times smaller after three cycles compared to that with the conventional electrolyte. These results clearly demonstrate that the use of a fluorinated electrolyte in DHC-LCP cells significantly enhances the electrochemical performance, emerging as a promising candidate for high-performance LIBs, providing both higher capacity utilization and longer cycle life.

## 4 Conclusions

Nanostructured LiCoPO_4_ (LCP) microspheres were successfully synthesized via one-step spray pyrolysis with the addition of diammonium hydrogen citrate (DHC) additive. Physical characterizations, including XRD, FTIR, XPS, SEM, and N_2_ adsorption–desorption analysis, confirmed the formation of an orthorhombic olivine structure with nanostructured morphology and high specific surface area attributed to the dispersion effect and evolution of the ammonium group of DHC during the pyrolysis process. Consequently, the initial capacity significantly improved to 132 mA h g^–1^, from 103 mA h g^–1^ without additives and 123 mA h g^–1^ with citric acid additive at 0.1 C with a conventional electrolyte. Furthermore, introducing a fluorinated electrolyte further enhanced the electrochemical performance, with initial and 50th discharge capacities of 141 and about 100 mA h g^–1^, respectively, at 0.1 C. These results highlight the potential of tailored additives in improving the properties of battery materials.

## Supporting Information Available

Characterization: detailed XRD patterns of bare-LCP, CA-LCP, and DHC-LCP samples; Rietveld refinement of bare-LCP and DHC-LCP samples; composition obtained by XPS spectra of LCP samples; TG-DTA curves of DHC; digital images of Li metal after cycling (PDF)
