# Supplementary material for: Diammonium Hydrogen Citrate-Assisted Spray Pyrolysis Synthesis of Nanostructured LiCoPO4 Microspheres as High-Voltage Cathode Material for Lithium-Ion Batteries
Source: ACS Omega. 2024 Sep 13;9(38):39596–603. doi: 10.1021/acsomega.4c03752 (PMC11425712; doi:10.1021/acsomega.4c03752)
Supplement: Supplementary file 1 — ao4c03752_si_001.pdf [file ao4c03752_si_001.pdf]

Diammonium hydrogen citrate-assisted spray pyrolysis synthesis of nanostructured $LiCoPO_4$ microspheres as high-voltage cathode material for lithium-ion batteries

Ayaulym Belgibayeva[1,2], Takeru Nagashima[1], Wenyu Cui[1], Daiki Sueyoshi[1], Izumi Taniguchi[1,*]

[1] Department of Chemical Science and Engineering, Tokyo Institute of Technology, Tokyo 152-8552, Japan

[2] National Laboratory Astana, Nazarbayev University, Kabanbay Batyr Ave. 53, Astana 010000, Kazakhstan

Corresponding Author*

E-mail: taniguchi.i.aa@m.titech.ac.jp (I.T.)

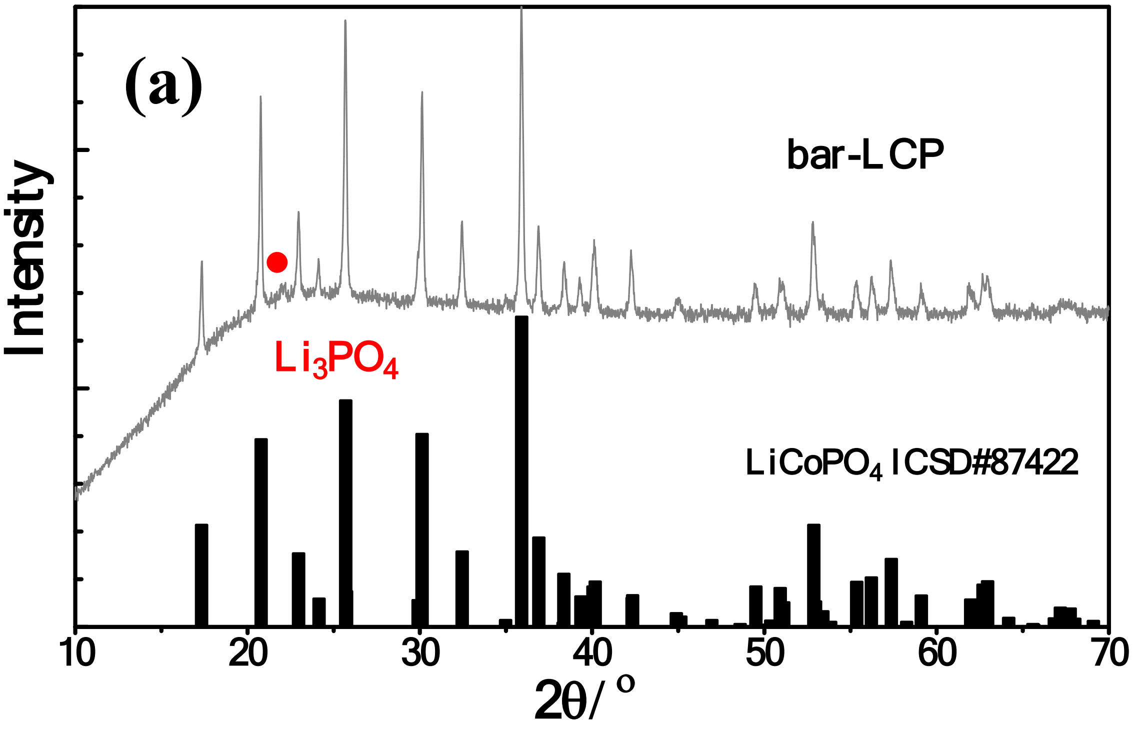
(a)
Intensity
bar-LCP
Li3PO4
LiCoPO4 ICSD#87422
10
20
30
40
50
60
70
2θ/ o

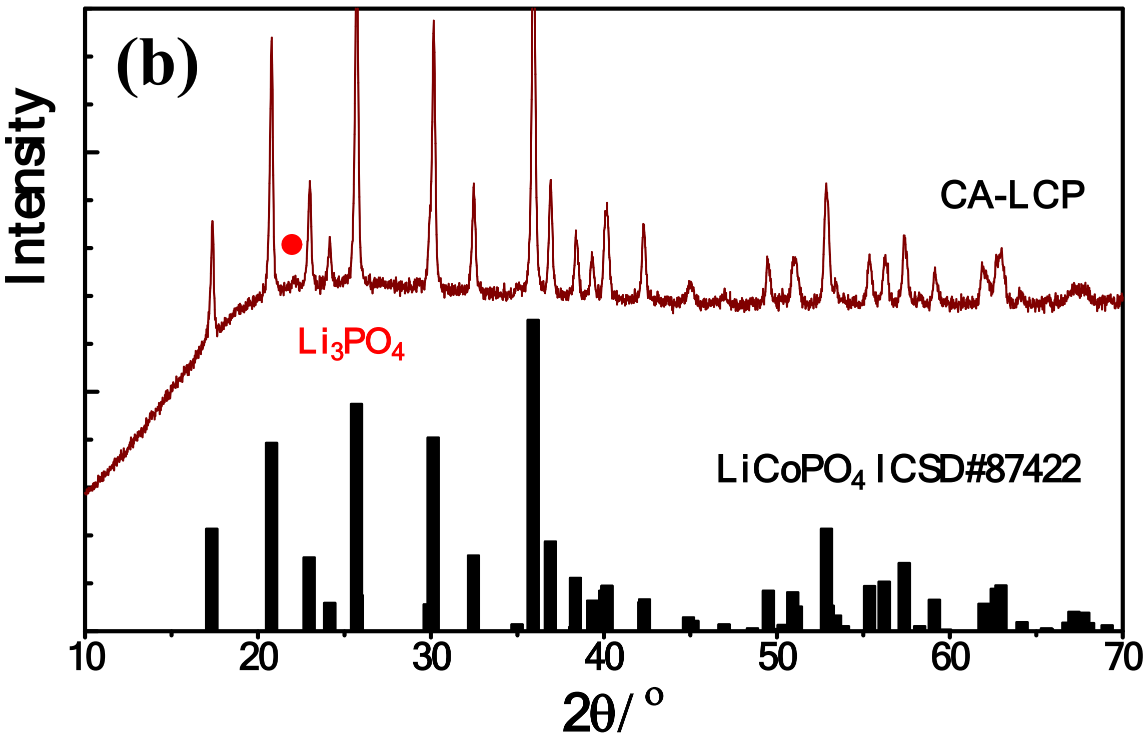
(b)
Intensity
CA-LCP
Li3PO4
LiCoPO4 ICSD#87422
10
20
30
40
50
60
70
2θ/ o

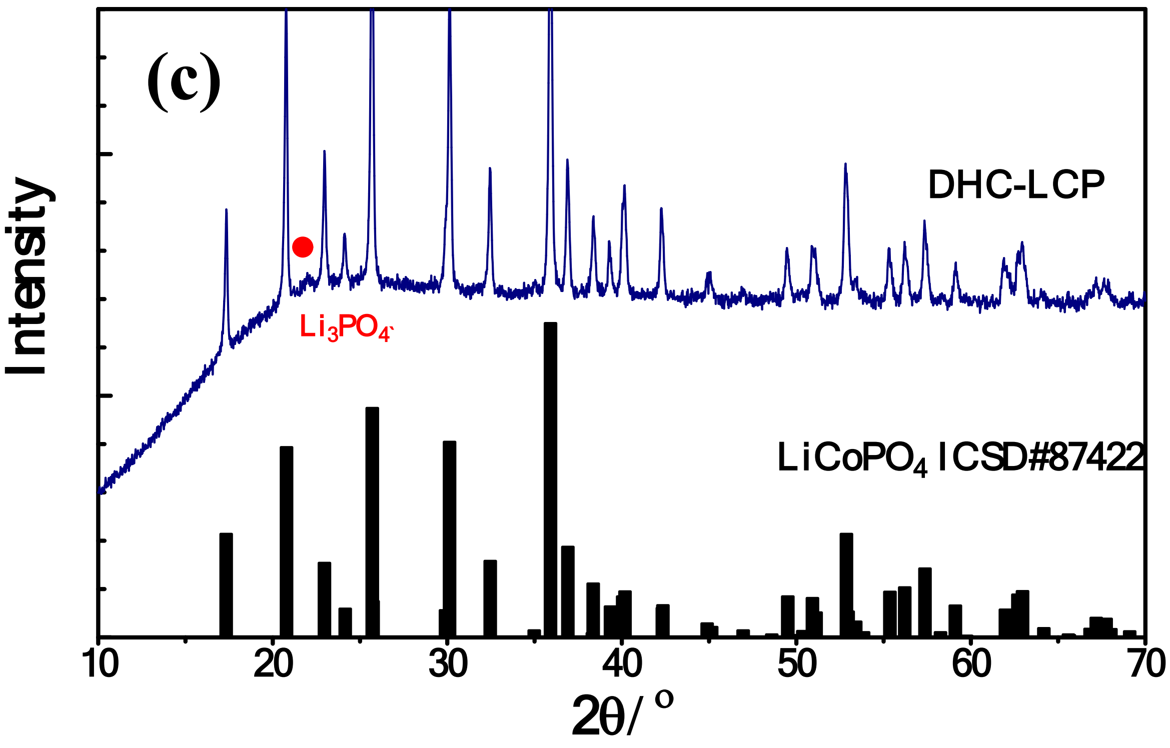

Figure S1 Details of the XRD patterns of bare-LCP (a), CA-LCP(b) and DHC-LCP (c).

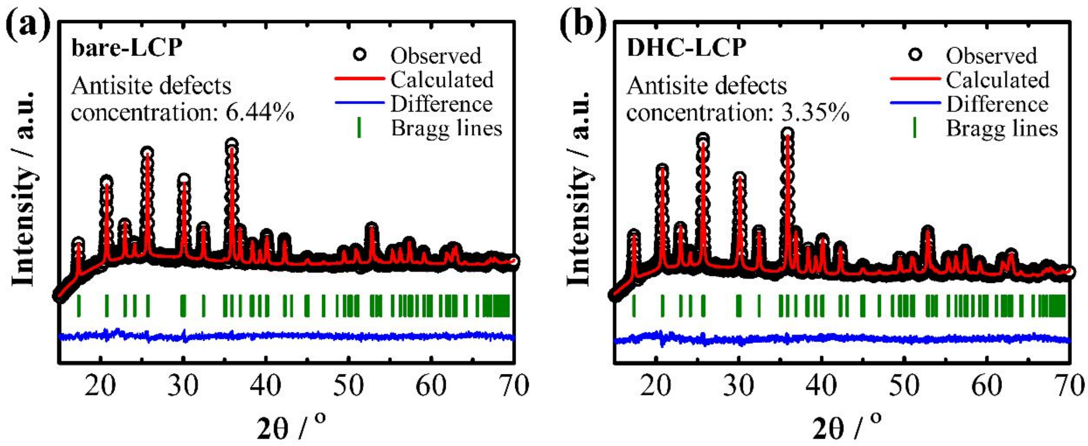

Figure S2 Rietveld refinement of XRD patterns of bare-LCP (a) and DHC-LCP (b).

Table S1 Composition of Co $2p_{3/2}$ XPS spectra of LCP samples

Sample	Co^{2+} (1) Peak position / eV	Co^{2+} (1) Ratio / %	Co^{2+} (2) Peak position / eV	Co^{2+} (2) Ratio / %	Satellite Peak position / eV	Satellite Ratio / %
Bare-LCP	781.2	18.2	782.5	31.0	786.2	50.8
CA-LCP	781.1	18.2	782.4	31.0	786.1	50.8
DHC-LCP	781.1	18.2	782.4	31.0	786.2	50.8

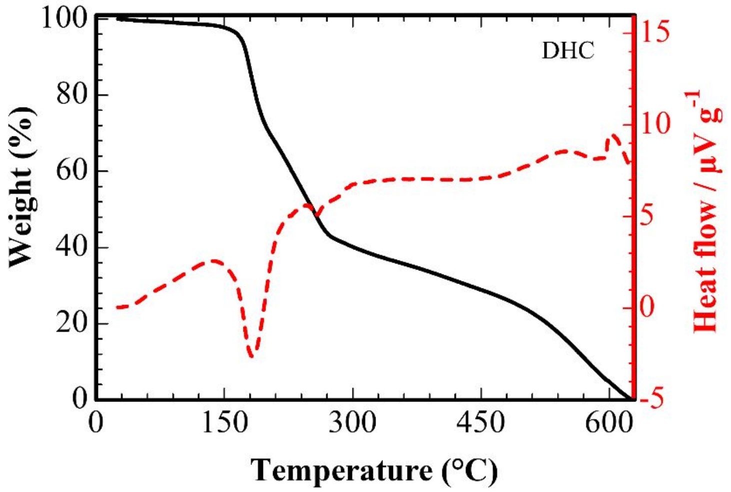

Figure S3 TG-DTA curves of diammonium hydrogen citrate (DHC) in air.

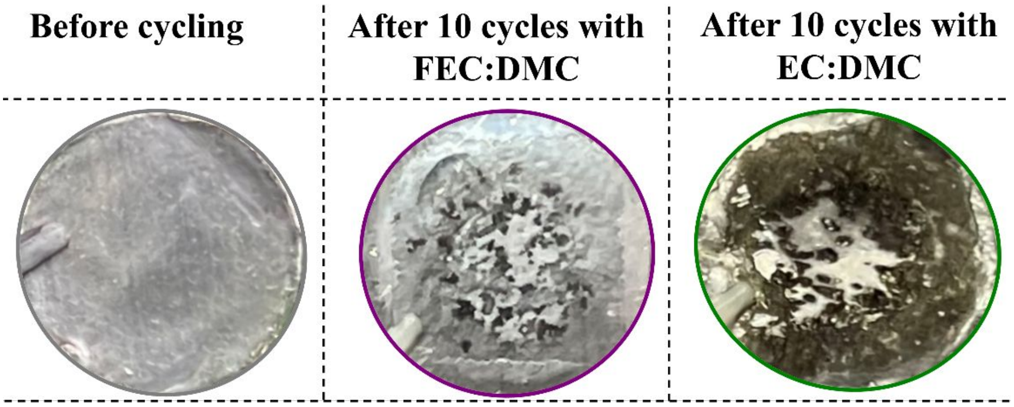

Figure S4 Digital images of the surface of Li metal (d = 12 mm) before and after 10 cycles at 0.1 C with DHC-LCP and different electrolytes.
